# Supplementary material for: Modified Crosstalk between Phytohormones in Arabidopsis Mutants for PEP-Associated Proteins
Source: Int J Mol Sci. 2024 Jan 27;25(3):1586. doi: 10.3390/ijms25031586 (PMC10855609; doi:10.3390/ijms25031586)
Supplement: Supplementary file 1 [file ijms-25-01586-s001.zip › ijms-2813658-supplementary.pdf]

Supplementary data

Modified Crosstalk between Phytohormones in Arabidopsis Mutants and PEP-Associated Proteins

Ivan A. Bychkov[1], Aleksandra A. Andreeva[1], Radomira Vankova[2], Jozef Lacek[2], Natalia V. Kudryakova[1]*, Victor V. Kusnetsov[1]

[1]Timiryazev Institute of Plant Physiology, Russian Academy of Sciences, Botanicheskaya 35, Moscow, 127276, Russia.

[2]Institute of Experimental Botany, Academy of Sciences CR, Rozvojova 263, 165 02 Prague 6, Czech Republic

Table S1. Inhibition of hypocotyl length (mm) in *pap* mutants and WT plants grown in darkness for 4 days on MS media supplemented with various concentrations of phytohormones. For each assay, three independent biological replicate experiments were conducted with 20 seedlings for each experiment.

**Treatment**	**Columbia 0, lengths (mm)**	***pap1*, lengths (mm)**	***pap6*, lengths (mm)**
**MS**	15.2±1.61	14.2±1.25	15.36±1.96
**metJa 5×10^{-6} M**	16.0.± 1.07	12.8±2.2	13.73±3.08
**metJa 10^{-5} M**	15.3±1.57	13.2±1.75	13.0±2.87
**metJa 5×10^{-5} M**	11.8±0.92	10.5±1.58	10.55±1.21
**SA 10^{-5} M**	17.4±1.35	16.4±1.26	14.5 ±2.34
**SA 5×10-^{5} M**	12.8±0.79	12.4±1.43	14±1.76
**SA 10^{-4} M**	11.5±1.35	11.8±0.79	11.8±1.40
**IAA 10^{-7} M**	14.3±1.16	15.3±1.42	15.6±1.71
**IAA 10^{-6} M**	10.8±1.25	10.1±2.13	10.8±1.23
**IAA 10^{-5} M**	10±2.1	9.6±2.23/6.3	9.9±1.96
**ACC10^{-7} M**	12.32±1.20	12.55±1.58	12.7±1.2
**ACC10^{-6} M**	7.79±1.18	7.59±1.14	8.58±1.62
**ACC10^{-5} M**	3.22±1.44	2.46±0.72	3.7±2.1

Table S2. Primary root growth inhibition in response to exogenous hormones. Seedlings were grown on MS media for 7 days under 16 h light/8 h dark conditions, subsequently transferred to vertical media plates containing a range of hormone concentrations and grown for another 4 days. Measurements were performed in three independent biological replicate experiments with 20 seedlings for each experiment.

**Treatment**	**WT, root growth (mm)**	***pap1*, root growth (mm)**	***pap6*, root growth (mm)**
**MS**	31.5±3.4	13.2±4.2	13.8±2.1

**SA10^{-7} M**	23.4±3.4	7.9±4.3	7.9±3.7
**SA 10^{-6} M**	18 ±3.4	4.5±1.8	5.0±2.9
**SA 10^{-5} M**	7.8±3.3	1.6±0.9	1.4±0.5
**metJA 10^{-7} M**	22.7±3.2	3.4±0.9	4.5±2.2
**metJA 10^{-6} M**	17.9±3.1	1.9±1.1	2.3±0.7
**metJA 10^{-5} M**	10.8±1.7	2.3±0.7	2.0±1.1
**ABA 10^{-6} M**	14.7±2.9	7.9±3.2	6.1±3.3
**ABA 10^{-5} M**	10.8±1.4	3.0±1.8	2.4±1.4
**ABA 10^{-4} M**	3.7±1.9	2.5±2.2	1.9±1.4
**ACC10^{-7} M**	21.7±1.3	4.6±1.5	5.8±3.3
**ACC10^{-6} M**	14.8±2.7	2.5±1.6	3.0±1.5
**ACC10^{-5} M**	7.1±2.6	1.4±0.40	0.9±0.7
**IAA 10^{-7} M**	15.3±1,5	4.9±2.1	4.9±1.2
**IAA 10^{-6} M**	4.9±0.4	2.2±1.13	2.1±0.8
**IAA 10^{-5} M**	2.5±9.6	1.1±1.8	1.5±0.7

Table S3. Sensitivity to hormone treatment during dark-induced senescence. The total protein content was measured in detached leaves (the 3d and 4th layer) of *A. thaliana* WT plants and *pap* mutant plants incubated in the dark at 23 °C in water or in solution of hormones for 3 days. Three biological replicates of each assay were performed with three technical replicates and the results were averaged. Asterisks indicate significant differences from the respective mock controls at p< 0.01 (Student's t test).

**Treatment**	**WT, protein content (μg/mm^2)**	***pap1*, protein content (μg/mm^2)**	***pap6*, protein content (μg/mm^2)**
**Initial**	0.577±0.049	0.332±0.063	0.374±0.008
**H2O**	0.439±0.042	0.237±0.049**	0.224±0.03**
**ACC 10^{-5} M**	0.370±0.013	0.154±0.008	.201±0.013
**ABA 5x10^{-5} M**	0.358±0.040	0.211±0.021**	0.207±0.073**
**IAA 10^{-6} M**	0.392±0.026	0.265±016	0.214±0.024
**MetJA 5x10^{-5} M**	0.363±0.017	0.158±0.016	0.173.2±0.014
**SA 10^{-5} M**	0.395±0.026	0.244±0.016	0.253±0.028

Table S4. Effect of hormone treatment on the relative expression levels of genes related to the chloroplast transcription machinery and chloroplast encoded genes. WT and mutant plants were grown on MS media in Petri dishes for four weeks under a 16 h light/8 h dark photoperiod at 23°C with 100 μmol·m^{-2}.s^{-1} and treated with solutions of hormones or an equal aliquot of ethanol for 3 h. Total RNA was analyzed via relative quantitative RT–PCR using *UBQ10* and *PP2A* as internal standards. The data presented in the table are the mean values (n ≥ 3). Error bars represent SEs. Different letters denote statistically significant differences between variants within the same genotype at p < 0.05 (ANOVA with post hoc Tukey's multiple-comparison test); asterisks indicate statistically significant differences between the control variants of the mutants

and the wild type at $p < 0.05$ (t test). At least three independent biological replicates and three technical replicates were used for the experiments.

Plants	MS	IAA	ACC	ABA	MetJa	SA
RPOTp						
**WT**	1.000±0.099^{b}	1.453±0.125^{a}	0.944±0.097^{b}	0.469±0.055^{c}	0.605±0.085^{c}	0.870±0.096^{bc}
pap1	0.743±0.076^{b*}	1.226±0.135^{a}	0.776±0.097^{b}	0.399±0.044^{c}	0.620±0.069^{b}	0.595±0.061^{b}
pap6	0.496±0.081^{ab*}	0.886±0.101^{a}	0.408±0.097^{b}	0.369±0.047^{b}	0.590±0.052^{ab}	0.565±0.049^{ab}
CKA4						
**WT**	1.000±0.079^{b}	0.892±0.096^{b}	0.961±0.093^{b}	1.731±0.191^{a}	1.268±0.137^{b}	1.119±0.130^{b}
pap1	1.945±0.223^{b*}	1.707±0.199^{b}	1.479±0.162^{b}	2.689±0.302^{a}	1.810±0.195^{b}	1.794±0.201^{b}
pap6	1.580±0.167^{b*}	0.922±0.108^{cd}	0.762±0.088^{d}	2.090±0.215^{a}	1.018±0.098^{c}	1.617±0.158^{b}
SIG1						
**WT**	1.000±0.123^{b}	2.005±0.227^{a}	1.404±0.151^{b}	0.452±0.049^{d}	0.705±0.080^{d}	0.651±0.072^{cd}
pap1	0.804±0.091^{b}	1.312±0.140^{a}	1.351±0.124^{a}	0.682±0.073^{bc}	0.602±0.071^{c}	0.592±0.058^{c}
pap6	0.901±0.094^{b}	1.710±0.188^{a}	1.392±0.129^{a}	0.653±0.067^{bc}	0.421±0.039^{c}	0.531±0.054^{c}
SIG2						
**WT**	1.000±0.100^{b}	1.811±0.209^{a}	1.597±0.154^{a}	0.650±0.071^{c}	0.953±0.110^{b}	1.066±0.124^{b}
pap1	1.490±0.152^{b*}	2.340±0.265^{a}	1.911±0.201^{ab}	0.728±0.077^{c}	1.164±0.188^{b}	1.613±0.172^{b}
pap6	1.081±0.119^{b}	1.612±0.177^{a}	1.556±0.161^{a}	0.636±0.069^{c}	1.052±0.091^{b}	1.307±0.128^{ab}
SIG3						
**WT**	1.000±0.073^{b}	1.809±0.201^{a}	1.597±0.160^{a}	0.349±0.041^{c}	0.414±0.049^{c}	0.511±0.056^{c}
pap1	0.519±0.062^{b*}	0.983±0.087^{a}	1.103±0.119^{a}	0.386±0.035^{b}	0.498±0.052^{b}	0.391±0.042^{b}
pap6	0.786±0.084^{b}	1.351±0.142^{a}	1.421±0.163^{a}	0.510±0.046^{c}	0.476±0.051^{c}	0.804±0.096^{b}
SIG4						
**WT**	1.000±0.116^{b}	2.108±0.219^{a}	1.452±0.138^{b}	0.403±0.045^{c}	0.688±0.074^{c}	1.225±0.131^{b}
pap1	0.604±0.065^{c*}	1.617±0.184^{a}	1.111±0.124^{b}	0.251±0.020^{d}	0.372±0.036^{d}	0.856±0.090^{bc}
pap6	0.691±0.077^{b*}	1.652±0.181^{a}	1.306±0.145^{a}	0.290±0.031^{c}	0.411±0.040^{c}	1.301±0.142^{a}
SIG5						
**WT**	1.000±0.147^{c}	0.272±0.023^{c}	1.534±0.160^{b}	4.823±0.509^{a}	0.757±0.082^{d}	0.637±0.072^{d}
pap1	1.819±0.204^{b*}	0.503±0.061^{d}	2.144±0.228^{b}	5.979±0.614^{a}	1.619±0.171^{b}	1.005±0.092^{c}
pap6	2.019±0.215^{b*}	0.339±0.0.40^{d}	2.638±0.291^{b}	7.817±0.826^{a}	1.849±0.205^{b}	1.036±0.118^{c}
SIG6						
**WT**	1.000±0.130^{b}	1.911±0.202^{a}	1.202±0.125^{b}	0.410±0.043^{d}	0.382±0.039^{d}	0.653±0.071^{c}
pap1	0.839±0.082^{b}	1.666±0.171^{a}	1.295±0.133^{a}	0.325±0.038^{c}	0.491±0.054^{c}	0.727±0.075^{b}
pap6	0.632±0.058^{b*}	1.598±0.156^{a}	1.113±0.120^{a}	0.219±0.024^{c}	0.302±0.036^{c}	0.602±0.067^{b}
PAP1						
**WT**	1.000±0.109^{a}	1.411±0.128^{a}	1.182±0.126^{a}	0.352±0.036^{b}	0.427±0.045^{b}	0.529±0.051^{b}
pap6	1.406±0.138^{c}	3.410±0.381^{a}	2.414±0.237^{b}	0.713±0.081^{d}	0.914±0.089^{d}	0.850±0.087^{d}
PAP6						
**WT**	1.000±0.116^{b}	1.962±0.202^{a}	1.400±0.139^{b}	0.522±0.049^{c}	0.613±0.060^{c}	0.907±0.088^{b}
pap1	1.802±0.195^{b*}	2.467±0.239^{a}	2.543±0.288^{a}	0.710±0.073^{c}	0.981±0.104^{c}	1.213±0.117^{c}
rpoB						
**WT**	1.000±0.104^{a}	1.113±0.108^{a}	1.152±0.120^{a}	0.848±0.079^{a}	0.880±0.093^{a}	0.951±0.102^{a}
pap1	0.851±0.086^{a}	1.761±0.179^{a}	0.765±0.081^{b}	0.673±0.073^{b}	0.680±0.063^{b}	0.678±0.079^{b}
pap6	0.802±0.091^{b}	1.531±0.163^{a}	0.682±0.066^{b}	0.649±0.066^{b}	0.673±0.060^{b}	0.593±0.064^{b}
clpP						
**WT**	1.000±0.113^{a}	1.102±0.102^{a}	1.209±0.149^{a}	0.852±0.091^{a}	0.896±0.084^{a}	0.821±0.086^{a}
pap1	2.412±0.271^{a*}	2.894±0.306^{a}	1.953±0.201^{b}	1.096±0.108^{c}	0.893±0.091^{c}	1.688±0.173^{b}
pap6	2.103±0.221^{ab*}	2.418±0.255^{a}	1.766±0.188^{b}	0.978±0.085^{c}	0.959±0.099^{c}	1.575±0.161^{b}
accD						
**WT**	1.000±0.094^{a}	1.061±0.108^{a}	1.217±0.118^{a}	0.811±0.076^{a}	0.857±0.088^{a}	0.864±0.089^{a}
pap1	3.117±0.333^{a*}	3.740±0.391^{a}	2.836±0.298^{b}	1.277±0.130^{c}	1.422±0.136^{c}	2.461±0.261^{b}
pap6	2.809±0.297^{a*}	2.339±0.240^{a}	2.612±0.277^{a}	1.202±0.116^{b}	1.209±0.133^{b}	2.133±0.225^{a}

Table S5. List of primers used for quantitative RT–PCR analysis.

Gene name	Locus	Forward primer (5′-3′)	Reverse primer (5′-3′)
rpoB	ArthCp014	ATG AGC AAC ACC AAA CCC CT	GGG TAG GCG AAA TGG AGG TT
accD	ArthCp031	GCT ACC AAT CAA TGT TTA CCT C	GAT TGA TAA TCA CAT AAA ACC G
clpP	ArthCp048	CAT TCC AGA TAT TAC CCA TCC A	GCC AAG AGG TTG ATA CCG AA
RPOTp	At2g24120	TTG CTG CTG CTT GCT ATT CTG C	GCA CAA TCA CCA AGC CAA CT

cPCK2	AT2G23070	TCT TTT ATG GCC ATG ACA ACT	CTG TTG GCC TTT CTT GGT
SIG1	At1G64860	CAT TGC GGA TAC TCG TTT GG	CCC GTT CTC CGA GTG TTG C
SIG2	At1G08540	TCT TCT TCG TCT TCA TCA TCC GC	CTG CTG CTG CTA CAA CTA CTG CTT
SIG3	At3G53920	GGA GGT CGA GTG GAC AGC T	TTC TCT CCA TGT CCG CCA CT
SIG4	At5G13730	TCA CGA GGA TTC AGA GAG GTA	GCT ATC AAC CAC TCT ATC CAC TG
SIG5	At5g24120	AGA TGT TGA TGG TGT TGG AGC	GAC TCT CTT TCG GCT TCA ATG
SIG6	At2g36990	TCG CCT ATT GTT GGT TCG C	GGG CTG ATA ATG ATG ATG CG
PAP1	At3g04260	TGA GAG TGG TGG AGA GAT TAC GGA A	CCA TCT TGC TTG CTG CTT TCG
PAP6	At3g54090	GGC GGA GAC TGT GAA AGA ACC A	GTA TTG CCG TAA AAA GAG TTG ATT GC
LOX2	At3g45140	GTA CGT CTG ACG ATA CC	TCT GGC GAC TCA TAG AA
AOS	At5g42650	CCA TAC ATT TAG TCT ACC AC	GCT AAT CGG TTA TGA ACT TG
Thi2.1	At1g72260	CTC AGC TGA TGC TAC CAA TGA GC	GCT CCA TTC ACA ATT TCA CTT GC
PDF1.2	At5g44420	TGT TCT CTT TGC TGC TTT CGA CG	GCA TGA TCC ATG TTT GGC TCC T
ICS1	At1g74710	CCC GCA AGA AGT ATG AGT CAT GT	AGT TCA GAG ACG GCG GAG ATT AG
NPR1	At1g64280	AGG CAC TTG ACT CGG ATG ATA TTG	CTT CAC ATT GCA ATA TGC AAC AGC
PR1	At2g14610	TTC ACA ACC AGG CAC GAG GA	GCA GCG TAG TTG TAG TTA GCC TTC
SAUR21	At5g01830	ATC CGC TCA TCT CTG CTT CG	AGG TCA AAG TCA TGG AGC GG
SUR1	At2g20610	GCT CGA CCA AAC GCA AAC AT	TGT TCT CGT CTG CAA TGG CT
GH3.9	At2g47750	GGT CGT CGT GGA AAG GGA TT	ATT GAG CCC GTC ACT ACT GC
IAA19	At3g15540	GAG ATG TGG CAG AGA AGA TG	TTC CTC AAA TAA GGC ACA CC
ACS4	At2g22810	ACA TGA GAC CTC TCC TTA GA	CCA GTT AGA GAC ATT TGA CA
ACO3	At2g05710	GAC CCA GAA AGA AGG AAA CAG G	AAT GTC TCA ACC ACA GCC ACC
ACS8	At4g37770	GGT TTT CCG GCT ATC GTT TCA	CAC ACT GCA TTA TCC GTT ACA
ERF1	At3g23240	ATT AGG GTT TGG CTC GGG AC	GAC TCT TGA ACT CTC TCC GCC G
ERF14	At1g04370	GGA TCA AGG AGG TCG TAG CAG TGG	TTA TTG CCT CTT GCC CAT GTT G
RD29	At5g52300	GTG AGA GGT GGT GTA ACG G	GTC GGT GCC TCT CTT TTC G
ABI4	At2g40220	CAA GGA GGA AGT GGG TGT AAT AAT AA	ATC CAG ACC CAT AGA ACA TAC CG
CYP7071a	AT4G19230	CGT GTC CTG GAA ATG AAT TAG CC	GCA CAA TGG GCA GTC CGT TT
NCED4	At4g19170	CAC CGA AAC TCC GAC CAG AAA	CGG AAG GAC GTG AAG GTG GAT
ABA2	At1g52340	GCG GTA CAT AAG CGG AGA TA	GAC ATG ATA AAT TGG CGG ACA

UBQ10	At4G05320	GCG TCT TCG TGG TGG TTT CTA A	GAA AGA GAT AAC AGG AAC GGA AAC A
Phos2a	At3g25800	ACT GCA TCT AAA GAC AGA GTT CCA	CCA CAA GCC CAG GAC GAA T
